# The Woman or the Man?

**Published:** 1891-11-28

**Authors:** 


### The Doctor's Armchair.

THE WOMAN OR THE MAN?

"Moral Bacilli" is an effective cross-heading for a sensational paragraph on the weaknesses and follies of charitable people. Mrs. Lynn Linton, with her usual wealth of epithet, treats the readers of the *Daily Graphic* to a piece of all-round scolding of the men and women whose hearts may be kinder, but whose heads, she thinks, are weaker than her own. The good lady appears tc be under the delusion that she is scientific. If she were a man instead of a woman one would have no hesitation whatever in pronouncing her merely silly. She seems to think that she has got a firm grip of the doctrine of the survival of the fittest; and, like the man with the apple-dumpling, she is convinced that "it is the jockey for her." One would like to suggest a little more expansive and profound study of the subject to Mrs. Lynn Linton before she makes another public appearance in this character. If she will condescend to take the word of a mere man, she may assure herself that such subjects as the doctrine of the survival of the fittest do not lend themselves easily and naturally to the "washing-tub" tactics of putting your hands on your hips and perorating at large.

Says this feminine apostle of afternoon-tea barbarism: "There is no question but that thousands of children are kept alive whom it would be charity to themselves and justice to the community to allow to die. Nature means them to die. They are her failures, her abortions, of no more value to the world than the dwarfed undergrowth of wayside weeds. But they are human beings. Born though they are to disease, vice, crime, pauperisation—destined to be the scourges of their generation so far as their influence can reach, and known to be so destined, our pity carries us over every other consideration, and we pour out all the treasures we can command—wealth, science, care, thought—to keep alive creatures that will be of no happiness to themselves nor of use to others. They are the parasites of society, sucking out its strength and contributing nothing to its well-being—moral bacilli creating disease and spreading death on all they contaminate with their presence."

It is plain from this little demonstration that Mrs. Lynn Linton is a "nice" woman. But it is equally evident that speech and not thought is her strong point. So far as science and reasoning power are concerned, she is clearly of the weaker sex For if she had been a man, she would have had some regard for her scientific and literary reputation; and if she had had a proper regard for her reputation, she would have asked herself a few questions before putting her name to an article which deals with some of the profoundest scientific, moral, and economic problems which are open to the investigation of man. Supposing Mrs. Lynn Linton had given herself the trouble to take but a cursory glance over the past history and experience of the human race, what would she have found? She would have found that a great many races and peoples have carried out the very principles she contends for. The doctrine of the survival of the fittest has been the primest of all the articles of their faith and practice. They have not only allowed their weakly offspring to die, but they have lent to busy death a helping hand by all the various methods of infanticide.

Now Mrs. Lynn Linton, if she had worked out this little detail of elementary science, would soon have come to a just conclusion about the value of her own doctrines, for she would have compared the savage races who have lived by the doctrine of the survival of the fittest with those civilised peoples who have accepted as part of their living faith the doctrine of what she calls the "divine pity," and the "grand brotherhood of Christianity." But whether Mrs. Lynn Linton has or has not taken the trouble to compare the Christian races with the savage, in material resources, intellectuality, morality, general elevation of character, people of sober sense can make the comparison for themselves. If the good woman, without having made the comparison, prefers the savage and his doctrines and practices, why does she not give effect to her convictions, and seek a stalwart lord among Jamieson's cannibals, and rear his and her own "dusky race" in the grand primal and elementary principles of which she is so fluent an apostle? When we find people giving practical effect to their convictions, as Laurence Oliphant and St. Paul did for example, we attach some importance to what they say. But when we hear one set of principles preached, and see quite another set practised, we do not usually consider the preacher as occupying any greater elevation than that of a mere kite-flyer.

But Mrs. Lynn Linton need not go so far afield as to Central Africa in order to find both a savage "lord" who will agree with her principles, and a theatre for putting them into practice. There are large areas in every populous town of her own country where not only are all the "unfittest" left to die in an easy or a painful manner, as the case may be, but many of the "fittest" also are gaily done to death, not to make a Roman holiday, but to make a three hours' orgie for a gin-drinking woman and a Lintonian philosopher of a man. Scientific pairs of this sort, being greedy of enjoyment, and having the power to purchase it by means of the insurance and death of their offspring, carry out their Lintonian principles to their logical issue, and furnish us with oft-recurring illustrations of the triumph of mind over matter, and of the victory of the strong over the weak: of, in fact, the survival of the brutallest.

As a sample of what goes on in London and other large towns in those districts where the fittest and the unfittest struggle together without let or hindrance from civilised methods, the following quotation from the annual report of a well-known society will be read with interest: "Most of the victims," says the report, "were young; many were babies, made habitually to feel the oppression of hatred, the dizziness of famine, and scarifying and curses, with blows and kicks and floggings with the oppressors' straps, pokers, ropes, boots, chairs, kettles and frying-pans; diggings into with prongs of fork and blade of knife; putting mustard oil into wounds; hanging up by the neck by a slip strap to a hook in the kitchen ceiling till black in the face and unconscious; thrusting a poker red-hot through the closed lips into the mouth, burning lips, tongue, and under the tongue; putting bare little thighs on top of hot ironing stove; making child grasp red-hot poker, beating with poker on the head, making, as the doctor called it, a "ring of bruises" completely round it, throwing sick child out of the window, breaking arm and leg; deliberately taking off comforting plaster-cast put on to little cripple at hospital, smashing it, throwing it under the bed, and leaving the puny creature to pine in pain again day and night; fixing big jaws of teeth in the fat of the thigh while child under bed for refuge, dragging it out, standing up with it, and shaking it 'as a dog shakes a rat'; flinging a baby across a room at a wall; immersing for half-an-hour, naked, in freezing tank, out of doors; tying, naked, to post in the yard, in the night; putting in yard for two hours, tied in chair, child with bronchitis; deliberately taking off splints newly put upon broken leg, and of wantonness, making child go about so; sending child about with broken arm, of malice to it; and cruel starvation when there was plenty, and imprisonments in attics and coal cellars for days, without so much as a drop of water."

That is Mrs. Lynn Linton's doctrine of the survival of the fittest as it works out in actual practice in England. Mr. Benjamin Waugh objects to the doctrine in these concrete forms; Mrs. Lynn Linton, of course, does not. Readers will choose between the two. For our own part, what strikes us is the exceedingly feeble intellectual grasp the amiable lady has of her own principles. The weak and sickly children whom she describes as "moral bacilli," "parasites" that suck the life out of the stronger organisms, should at least be dealt with scientifically. If this kind gentlewoman were fully abreast of the science of the times, she would not leave these "moral bacilli" to poison and destroy the organisms that are so unlucky as to be strong; she would call upon the State to organise an army of "moral phagocytes," in blue coats, and armed with truncheons, to knock the pestiferous little "unfittest" on the head. If she had a really strong mind as well as a "ranting" tongue, she would see that the proper scientific alternative to seeking out and restoring delicate children to health and life, is not leaving them alone to die slowly or quickly as they may, but hunting them up and drowning them like superfluous kittens, or putting an end to their wicked activities in a lethal chamber, as Dr. Richardson polishes off stray dogs.

Here space bids us stop; but we would like to close with the expression of the conviction that though Mrs. Lynn Linton is both a scientific woman and a philosopher in her own estimation, Mr. Benjamin Waugh, who makes no claim to be either, is, on the whole, to be preferred.

